# Editorial Department

**Published:** 1857-12

**Authors:** Wm. Henry Thayer, S. B. Hunt

**Affiliations:** Keene, N. H.; Keene, N. H.


KEENE, N. H., Oct. 16th, 1857.

DEAR SIR,

I was reading the proceedings of the Buffalo Medical Association in your very interesting Journal, (received yesterday,) I observed that in the long enumeration of indigestible articles swallowed, the gentlemen reporting them have overlooked an interesting collection of such cases described in J. B. S. Jackson's catalogue of the Cabinet of the Med. Improv't Soc. of Boston. you have a copy of that catalogue, you will find them Nos. 527 to 533, and including a penknife (open), silver pencil case, pieces of glass, nails, cotton, pieces of blanket, etc., the most of them by lunatics. The most remarkable, I think, that I ever heard of, is one of these, a piece of blanket 2½ feet long by 4 to 10 inches wide,—an article that it seems almost impossible to swallow. The glass, some of it in large pieces and irregular, caused no unpleasant symptoms—which renders the results of Spallanzani's experiments with fowls less remarkable, or rather which equals the strangeness of those.

Respectfully yours,

WM. HENRY THAYER.

S. B. HUNT, M. D.

